# Supplementary material for: Relative Influence of Land Use, Mosquito Abundance, and Bird Communities in Defining West Nile Virus Infection Rates in Culex Mosquito Populations
Source: Insects. 2022 Aug 23;13(9):758. doi: 10.3390/insects13090758 (PMC9502061; doi:10.3390/insects13090758)
Supplement: Supplementary file 1 [file insects-13-00758-s001.zip › Figure S1.pdf]

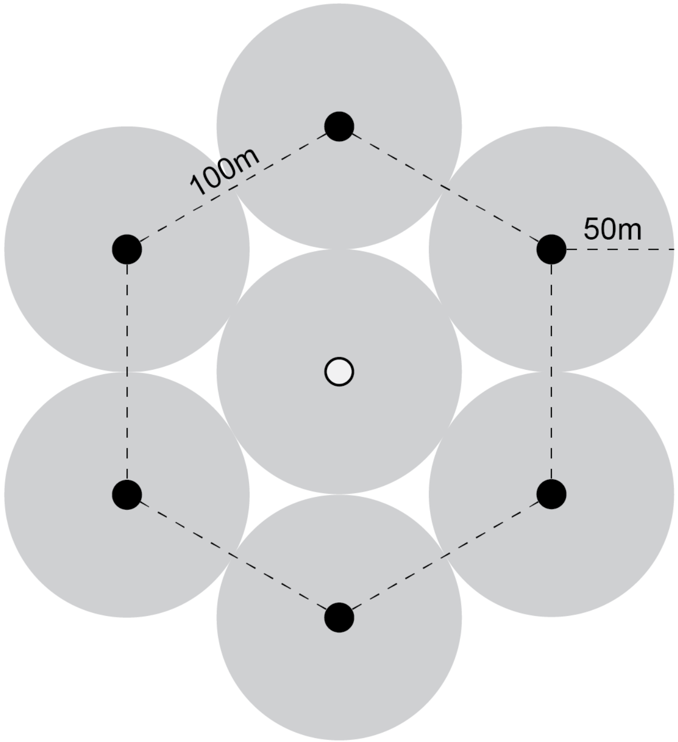

Figure S1. Overview of observation locations for bird surveys at each study site. Small circles indicate points at which an observer would stand, with the lighter circle representing the location nearest the mosquito trap; large, gray circles indicate the 50m-radii survey areas in which birds were counted around each point; dashed lines show distances between points and circle radii. Note that if a planned point location would put an observer in danger (e.g. falling in the middle of a busy road), that point's location was adjusted to fall further than 100m, but still within 150m, from neighboring points.
