# Supplementary material for: Relative Influence of Land Use, Mosquito Abundance, and Bird Communities in Defining West Nile Virus Infection Rates in Culex Mosquito Populations
Source: Insects. 2022 Aug 23;13(9):758. doi: 10.3390/insects13090758 (PMC9502061; doi:10.3390/insects13090758)
Supplement: Supplementary file 1 [file insects-13-00758-s001.zip › Table S1.pdf]

Table S1. Average mosquito community metrics, collected via NJ light traps, across sampling sites in central Iowa during 2016-2018.

	Culex pipiens group (CPG)			Culex spp.			All Mosquitoes			% *Culex pipiens* group			% *Culex* spp.		
Site	All Season	Early Season	Late Season	All Season	Early Season	Late Season	All Season	Early Season	Late Season	All Season	Early Season	Late Season	All Season	Early Season	Late Season
COGA	220	114.67	105.33	275.67	137	138.67	3704.67	2220	1484.67	5.94	5.17	7.09	7.44	6.17	9.34
EMMC	390.67	264	126.67	469	328.33	140.67	3925.33	2242.33	1683	9.95	11.77	7.53	11.95	14.64	8.36
EWIN	1440	980	460	1519	1005.67	513.33	20409.67	15263.67	5146	7.06	6.42	8.94	7.44	6.59	9.98
GRAN	277.33	73.33	204	317.67	80.33	237.33	4352	2301.33	2050.67	6.37	3.19	9.95	7.30	3.49	11.57
JEPA	725.33	151.67	573.67	810	166.67	643.33	12009	2579	9430	6.04	5.88	6.08	6.74	6.46	6.82
MOOR	181	87	94	221	106.5	114.5	3015	971.5	2043.5	6.00	8.96	4.60	7.33	10.96	5.60
WELK	1033.67	663.33	370.33	1215	759.33	455.67	19636	14154	5482	5.26	4.69	6.76	6.19	5.36	8.31
YEBA	151	44	107	165.5	48.5	117	11994.5	9775	2219.5	1.26	0.45	4.82	1.38	0.50	5.27
%CV	85.48	114.74	73.5	81.18	108.75	71.4	73.22	95.67	75.73	39.89	59.26	26.64	41.07	64.15	26.65
