# Supplementary material for: Relative Influence of Land Use, Mosquito Abundance, and Bird Communities in Defining West Nile Virus Infection Rates in Culex Mosquito Populations
Source: Insects. 2022 Aug 23;13(9):758. doi: 10.3390/insects13090758 (PMC9502061; doi:10.3390/insects13090758)
Supplement: Supplementary file 1 [file insects-13-00758-s001.zip › Table S2.pdf]

Table S2. Bird community metrics, from point counts, across sampling sites in central Iowa during 2018.

Site	Abundance (average total detections per visit)	Richness	Simpson's Diversity	Shannon Diversity	Cumulative Amplification Fraction
COGA	29.57	19	0.88	2.43	3.2
EMMC	13.57	23	0.91	2.73	0.31
EWIN	21.17	26	0.92	2.85	1.52
GRAN	24.57	25	0.88	2.63	2.69
JEPA	19.43	25	0.9	2.71	1.27
MOOR	23.57	24	0.77	2.23	1.13
WELK	24.86	28	0.92	2.82	1.52
YEBA	32	27	0.9	2.63	2.34
%CV	24.44	11.27	5.47	7.97	53.59
